# Sprint versus isolated eccentric training: Comparative effects on hamstring architecture and performance in soccer players

**DOI:** 10.1371/journal.pone.0228283

**Published:** 2020-02-11

**Authors:** Jurdan Mendiguchia, Filipe Conceição, Pascal Edouard, Marco Fonseca, Rogerio Pereira, Hernani Lopes, Jean-Benoît Morin, Pedro Jiménez-Reyes

**Affiliations:** 1 Department of Physical Therapy, ZENTRUM Rehab and Performance Center, Barañain, Spain; 2 Center of Research, Education, Innovation and Intervention in Sport, Faculty of Sports, University of Porto, Porto, Portugal; 3 LABIOMEP—Porto Biomechanics Laboratory, University of Porto, Porto, Portugal; 4 Inter‐university Laboratory of Human Movement Biology (LIBM EA 7424), University of Lyon, University Jean Monnet, Saint Etienne, France; 5 Department of Clinical and Exercise Physiology, Faculty of medicine, Sports Medicine Unity, University Hospital of Saint-Etienne, Saint-Etienne, France; 6 Medical Commission, French Athletics Federation (FFA), Paris, France; 7 Health and Performance Unit of Portuguese Football Federation, Portugal; 8 Clínica do Dragão, Espregueira-Mendes Sports Centre–FIFA Medical centre of Excellence, Porto, Portugal; 9 DEM-ISEP, School of Engineering, Polytechnic of Porto, Porto, Portugal; 10 Université Côte d’Azur, LAMHESS, Nice, France; 11 Centre for Sport Studies, Rey Juan Carlos University, Madrid, Spain; Universidade Federal de Mato Grosso do Sul, BRAZIL

## Abstract

**Aims:**

The purpose of this study was to compare the effects of hamstring eccentric (NHE) strength training versus sprint training programmed as complements to regular soccer practice, on sprint performance and its mechanical underpinnings, as well as biceps femoris long head (BFlh) architecture.

**Methods:**

In this prospective interventional control study, sprint performance, sprint mechanics and BFlh architecture variables were compared before versus after six weeks of training during the first six preseason weeks, and between three different random match-pair groups of soccer players: “Soccer group” (n = 10), “Nordic group” (n = 12) and “Sprint group” (n = 10).

**Results:**

For sprint performance and mechanics, small to large pre-post improvements were reported in “Sprint group” (except maximal running velocity), whereas only trivial to small negative changes were reported in “Soccer group” and “Nordic group”. For BFlh architecture variables, “Sprint” group showed moderate increase in fascicle length compared to smaller augment for the “Nordic” group with trivial changes for “Soccer group”. Only “Nordic” group presented small increases at pennation angle.

**Conclusions:**

The results suggest that sprint training was superior to NHE in order to increase BFlh fascicle length although only the sprint training was able to both provide a preventive stimulus (increase fascicle length) and at the same time improve both sprint performance and mechanics. Further studies with advanced imaging techniques are needed to confirm the validity of the findings.

## Introduction

Performing soccer-specific actions at high speed is a paramount physical feature of high-level soccer players. Short accelerations and linear sprints are two of the most important actions in soccer since they frequently precede goals and other decisive actions [1]. On the other hand, the majority of hamstring injuries (57%) occur during high-speed sprinting actions [2]. Specifically, hamstring muscle injuries (HMI) are the most prevalent injuries in soccer accounting for 12–16% of all injuries [3] and have not shown signs of clear decrease over the last three decades. Therefore, it seems logical to expect sprinting to be a key parameter in soccer both from a performance and injury point of view. Both the swing and the stance phase of sprinting, where the hamstring muscles are put under tension while lengthening (eccentric musculotendinous contraction) to decelerate knee extension have been suggested as possible scenarios of injury occurrence [4,5] and have laid the foundations of current prevention methods (eccentric strength training) of HMI in soccer [6].

Research suggests that this type of eccentric training results in a multifactorial adaptative response possibly including increases in motor unit discharge rate and changes in muscle architecture such as hypertrophy and fascicle lengthening [7–9]. Specifically, repeated exposure to lengthening hamstring contractions through the nordic hamstring exercise (NHE) seems to protect muscles from injury in soccer [6]. The main mechanism proposed by the authors is the increase in fascicle length, supposedly induced by an increase in the number of sarcomeres in series, which in turn results in less overall strain and also lower susceptibility to damage [10]. Moreover, Timmins et al. [8] have recently observed such an adaptation after eccentric knee flexor training on an isokinetic dynamometer while also noting that concentric training caused fascicle shortening, despite occurring at long muscle lengths. Furthermore, the same group of authors recently reported that soccer players with shorter biceps femoris long head (BFlh) fascicles (<10.56 cm on average) were at fourfold greater risk of hamstring strain injury than players with longer fascicles [11]. It is however important to keep in mind that clear methodological limitations are associated with ultrasound methodology to infer muscle fascicle lengths [12,13] as will be discussed below. Given the effectiveness of the predominantly eccentric NHE in increasing eccentric hamstring strength when added to soccer training [14], it is important to examine the impact of this single exercise on BFlh fascicle lengths concomitantly to real soccer practice and not only in isolated conditions.

In addition to its potential role in preventing posterior thigh muscle strains, hamstring muscle strength has been suggested as an important factor to improve sprinting performance in soccer as a horizontal force producer [15,16]. During the acceleration phase of sprinting, forward orientation of ground reaction force (GRF) has been shown to be the most powerful determinant of field sprint performance compared to the overall magnitude of vertical or resultant GRF [17]. Recently, Morin et al. [17] have shown that hamstring EMG activity during the swing phase and eccentric knee flexor peak torque were related to the amount of horizontal GRF produced during treadmill sprint accelerations converting the hamstrings as a key muscular determinant of sprint acceleration performance. These results suggest that the conjunction of hip extensors (hamstrings in particular) torque capability and degree of activation during the swing phase, is a key muscular determinant of sprint acceleration performance [17]. This important role of the posterior thigh muscles during sprinting could partly explain the altered capability to produce horizontal force at low speed during the first meters of the acceleration phase shown by soccer players after return to sport from a hamstring injury [18].

Different types of hamstring-focused strength training have been proposed in the literature to improve sprint performance in soccer players [14,16] but the direct, individual relationship between improvements in single joint hamstring strength and sprint performance and mechanics remains unclear. Interestingly, to date, no study exists about the effect of sprint practice (recently suggested as a potentially preventive method in adequate doses) [19,20], as a complementary training on the muscle architecture of soccer players. The velocity at which an athlete runs once at full speed or close is directly related to the velocity of the lower limb segments during the swing phase, and in turn the negative work done by the hamstrings [3] since they are significant contributors to human propulsion at very high speeds [17,21]. Furthermore, in addition to the significant length-tension sustained by the hamstring muscle-tendon unit during maximal velocity running [22], this specific exercise is the only one, by far, that elicits maximal levels of hamstring activity as assessed by surface electromyography [23,24]. It is expected that a comprehensive sprint training program may induce an overall improvement of sprint performance and underlying mechanical outputs, and BFlh structural adaptations associated to this eccentric-type overload for the muscle-tendon unit, including a greater fascicle length. Given the time constraint of modern soccer training, specific NHE or sprint training might not be systematically implemented [25], raising the question of their respective effectiveness as complementary interventions to the soccer training content.

The aim of this study was to compare the effects of hamstring eccentric (NHE) strength training versus sprint training programmed as complements to regular soccer practice, on sprint performance and its mechanical underpinnings, and BFlh architecture.

## Materials and methods

### Procedure

In this prospective interventional controlled study, sprint performance, mechanics and BFlh architecture variables were measured before and after six weeks of training during the first six preseason weeks in three different groups of soccer players. The “Soccer group” (controls) continuing their usual soccer practice, the “Nordic group” players performed a NHE program in addition to usual soccer practice, and the “Sprint group” performed a comprehensive sprint acceleration program in addition to usual soccer practice. All subjects were informed of potential risks associated with the experimental procedures before giving their written informed consent to participate and ethics approval was granted by the Faculty of Sports of the University of Porto, Portugal human research ethics committee, which conforms to the ethical standards established by the declaration of Helsinki.

### Participants

Soccer players were recruited from two different soccer teams playing in the same Elite Division of Football Association of Porto, North of Portugal. Within each team, players were randomly assigned to the different groups for the study. The possible effect of the training load on the study outcomes were mitigated by having all players proportionally distributed, and both teams following very close training and game programs and physical demands. Soccer teams were initially contacted and informed about the project via email. Inclusion criteria were: 1) to be older than 18 years; 2) to have a competitive experience in soccer for at least 3 consecutive years prior to measurements; and 3) to start the preseason at the scheduled time. Exclusion criteria were: 1) to be involved in any additional strength training program; 2) to present a history of hip, knee, or lumbo-pelvic joints injury in the past three years confirmed by MRI and that required intervention by a health professional, and 3) to suffer a neurological, cardiorespiratory or systemic disorder. 32 soccer players (16±6 per team) were voluntarily recruited and randomly assigned to either “Soccer group” (n = 10), “Nordic group” (n = 12), or “Sprint group” (n = 10). To reduce potential confounding, a match-pair design was used in which athletes were matched depending on their position (i.e., defender, midfield, and forward), playing status (i.e., starting or substitute player), and previous hamstring injury.

Nine players dropped out from the study: two from the “Soccer group” due to retirement form soccer and change to another club; five from the “Nordic group” due to a compliance of <80% to the training program (n = 3), one semitendinosus injury and one ankle injury; and two from the “Sprint group” due to both knee injury and adductor longus tear. All players trained four times per week during 90 minutes and played at least 180 minutes of friendly matches during the preseason period.

### Sprint performance and mechanics measurements

After a standardized warm-up, subjects performed two 50 m maximal sprints, separated by 6 min of passive rest, from a standing start on an artificial turf field with their habitual soccer boots. Tests were performed by the same investigator (FC), at the same time of the day (always before of their normal soccer training), under similar environmental conditions of temperature. Each sprint was measured by means of a Radar device with a 46.9 Hz sampling frequency (Stalker ATS II Version 5.0.2.1, Applied Concepts, Dallas, TX, USA), which was placed on a tripod 10 meters behind the subjects at a height of 1 meter corresponding approximately to the height of subjects’ center of mass [26,27]. From these speed–time measurements, a macroscopic biomechanical analysis-based on the laws of motion [28] was used to calculate the maximal horizontal external power (*P*_max_ (W·kg^-1^)), velocity (*v*_0_ (m·s^-1^)) and force (*F*_0_ (N·kg^-1^)) mechanical outputs during the acceleration. In addition, the ratio of force was calculated as the horizontal component of the ground reaction force divided by the resultant ground reaction force, and the maximal value of this ratio (*RF*_max_ (%)) was used as an indicator of the players ability to orient the ground reaction force in the forward direction at the beginning of their acceleration. The higher the *RF*_max_, the more forward the force orientation during the early phase of acceleration. Finally, sprint performance was described via the measurement of 5 m (s) and 20 m (s) times, as derived from the fitted speed-time curves (see [28] for more details) [28].

### Assessment of the BFlh architecture

BFlh muscle architectural characteristics has been performed using ultrasound following previously published procedure [29,30]. Muscle thickness (Thickness BFlh), pennation angle (PA) and the estimation of fascile length (FL) were determined from ultrasound images obtained along the longitudinal axis of the muscle belly using a 2D B-mode ultrasound (12 Mhz frequency, 8 cm depth; 14 x 47 mm field of view) (GE Healthcare Logiq S7, Wauwatosa, USA). The measurement site was the halfway point between the ischial tuberosity and the posterior knee joint fold, along the line of the BFlh. Once the scanning site was determined in each participant, several anatomical landmarks were taken (ischial tuberosity, fibula head and midpoint of the posterior knee joint fold) and photographs were taken in order to ensure reproducibility for future assessment sessions. All architectural measurements were performed after at least 5 minutes of inactivity, with the participant in prone position, with the hip in neutral position, and the knee positioned passively in full extension with their feet laying off the bed for comfort and they were instructed to remain relaxed during image acquisition. To obtain the images, the transducer was then aligned to the fascicle plane, which was assumed to correspond to the image with the most continuous and visible muscle fascicles (~25% or more of the total estimated length as a minimum) while the superficial and intermediate aponeuroses remained parallel (less than 4º between aponeuroses angle) in order to meet the stablished inclusion criteria [30]. For all scans, the probe was handled carefully by the sonographer (MF) and transmission gel was used to improve the acoustic contact and to keep the transducer pressure on the skin to a minimum [31].

**Fig 1:**
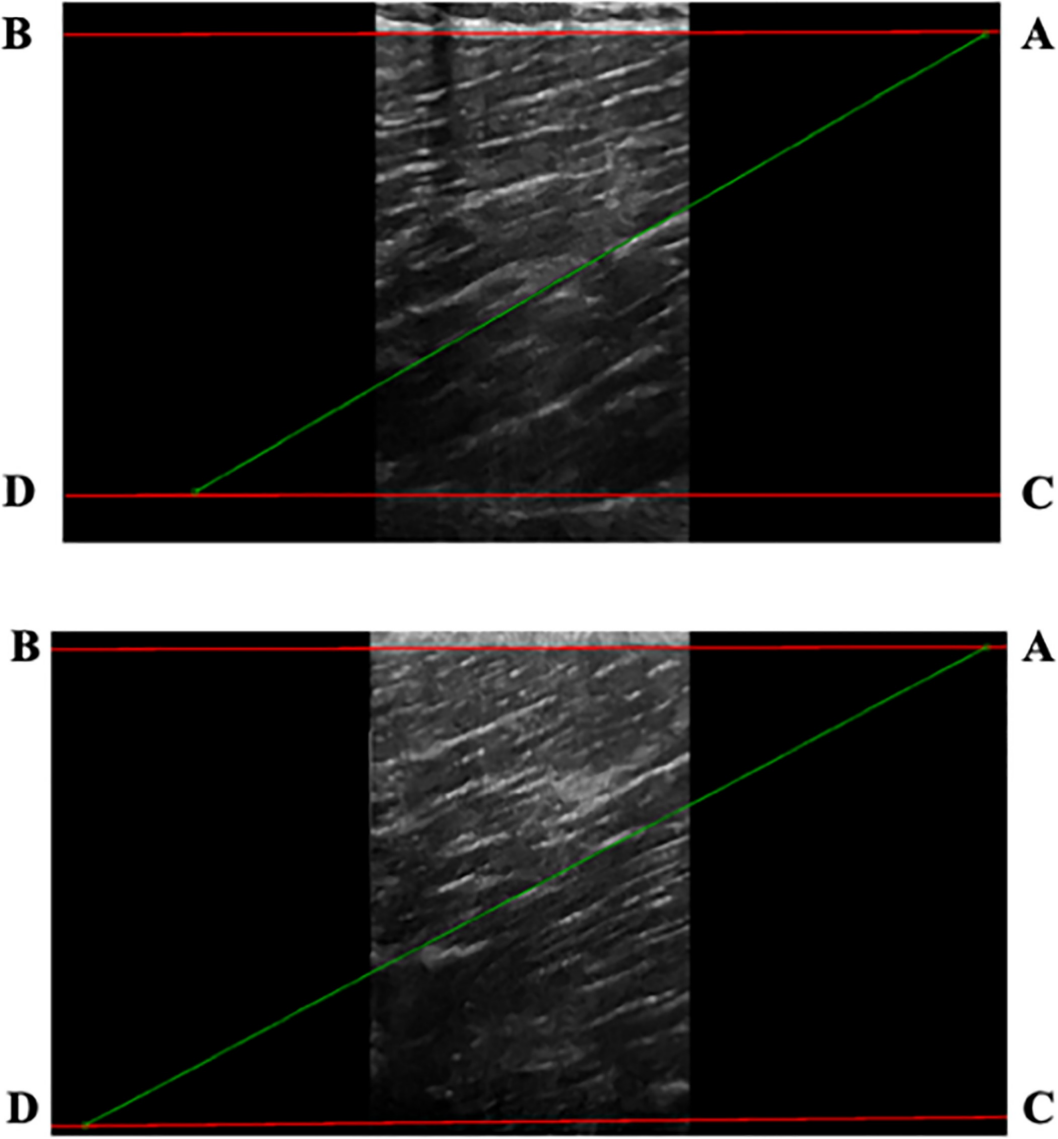
Two-dimensional ultrasound image of the pre-post intervention of Biceps Femoris long head (BFlh). In order to measure FL and PA, a line was placed along the length of a fascicle, which joined superficial aponeurosis (A-B line) and intermediate aponeurosis (C-D line). The FL was calculated as the length of this line. The PA was calculated as the angle between lines CD and DA.

After the scan, an analysis was carried out off line by means of a custom-made image processing routine developed in Matlab 2016a software, (The Mathworks, Inc., Natick, 2016). Fascicle length was measured by manually outlining visible parts of muscle fascicles and the sections that were not visible were extrapolated linearly to the linearly projected line of the aponeurosis [29] (see Fig 1). The angle between the line marking the intermediate aponeurosis and the outlined fascicle was the measured, giving the PA. MT was measured as the distance between the superficial and intermediate aponeuroses.

All images were collected and analyzed by the same researcher (MF), who was blinded to the identity and group of the participants during the analysis.

### Nordic exercise program

The NHE program was performed during six weeks only in the “Nordic group”. The NHE and the program was the same as the one proposed by Petersen et al. [6], but only completed over 6 weeks instead of the 10 weeks proposed in the original study. The exercise was conducted always after regular training sessions and players were supervised by their physical coach, who was informed about the exercise orally and had received written descriptions and illustrations of the exercise. Training program compliance and adverse effects were registered for each team on a weekly basis by contact with the coaches during the 6-week training period. A minimum of 48 h separated each training session. All training sessions were supervised by either researchers, physiotherapists or coaches.

### Sprint training program

The sprinting program was performed only by players assigned to the “Sprint group” during six weeks with two sessions of exercises per week separated by at least 72 hours (Table 1). Each session lasted about 30–35 min and the team head coaches decided when exactly the program was performed within the training session but they were advised to follow sprint training after a proper warm-up program. The first session of the week included sprint running exercises aiming at stimulating the entire force-velocity spectrum: normal sprint accelerations (5x30 m to 3x30 m), heavy sled sprints (sled load of 70% of body mass) from 1x10 m to 3x10 m, and 4x20 m (with 20 m run-up distance), and flying start sprints. The second session of the week included some ankle plantar flexors exercises with added load (from 50 to 70% body mass), unilateral bouncing exercises, plyometrics, and various athletics drills and acceleration exercises over short distances. All details are provided in Table 1.

**Table 1:** Training contents for the sprint training group.

	Weeks						
	1	2	3	4	5	6	7
**FVP Exercises**	**First training session of the week**						
	Very Heavy Sled (70% BW) 1x10-m	Very Heavy Sled (70% BW) 1x10-m	Very Heavy Sled (70% BW) 2x10-m	Very Heavy Sled (70% BW) 2x10-m	Very Heavy Sled (70% BW) 3x10-m	Very Heavy Sled (70% BW) 3x10-m	Very Heavy Sled (70% BW) 4x10-m
	Sprint 5 x 30-m	Sprint 5 x 30-m	Sprint 4 x 30-m	Sprint 4 x 30-m	Sprint 3 x 30-m	Sprint 3 x 30-m	Sprint 2 x 30-m
	Flying Run 4 x 20-m (20-m preparation)	Flying Run 4 x 20-m (20-m preparation)	Flying Run 4 x 20-m (20-m preparation)	Flying Run 4 x 20-m (20-m preparation)	Flying Run 4 x 20-m (20-m preparation)	Flying Run 4 x 20-m (20-m preparation)	Flying Run 4 x 20-m (20-m preparation)
**Gastro Exercises**	**Second training session of the week**						
	Gastrocnemius Extensions (50% BW) 2x6	Gastrocnemius Extensions (50% BW) 2x6	Gastrocnemius Extensions (60% BW) 3x6	Gastrocnemius Extensions (60% BW) 3x6	Gastrocnemius Extensions (70% BW) 2x6	Gastrocnemius Extensions (70% BW) 2x6	Gastrocnemius Extensions (70% BW) 2x6
	Gastrocnemius Rebounds (30% BW) 2x6	Gastrocnemius Rebounds (30% BW) 2x6	Gastrocnemius Rebounds (40% BW) 3x6	Gastrocnemius Rebounds (40% BW) 3x6	Gastrocnemius Extensions Unilateral (30% BW) 1x6	Gastrocnemius Extensions Unilateral (30% BW) 2x6	Gastrocnemius Extensions Unilateral (30% BW) 2x6
					Gastrocnemius Rebounds (50% BW) 3x6	Gastrocnemius Rebounds (50% BW) 2x6	Gastrocnemius Rebounds (50% BW) 2x6
					Gastrocnemius Rebounds Unilateral (10–20% BW) 1x6	Gastrocnemius Rebounds Unilateral (10–20% BW) 2x6	Gastrocnemius Rebounds Unilateral (10–20% BW) 2x6
**Acceleration Exercises**	**Second training session of the week**						
	Wall Acceleration Drill (2 steps) 3 x 5	Wall Acceleration Drill (2 steps) 3 x 6	Wall Acceleration Drill (4 steps) 2 x 6	Wall Acceleration Drill (4 steps) 2 x 7	Wall Acceleration Drill (4 steps) 2 x 8	Wall Acceleration Drill (4 steps) 2 x 6	Wall Acceleration Drill (2 steps) 3 x 5
	Free Sprint (10 m) 2 x 5	Free Sprint (10 m) 2 x7	Free Sprint (5 m) 2 x 4	10-m Weighted Sled Towing (15% BW) + 10-m Free Sprint 2 x 2	15-m Weighted Sled Towing (15% BW) + 10-m Free Sprint 2 x 2	15-m Weighted Sled Towing (15% BW) + 10-m Free Sprint 2 x 3	10-m Weighted Sled Towing (15% BW) + 10-m Free Sprint 2 x 2
		Free Sprint (20 m) 1 x 4	Free Sprint (10 m) 1 x 2	Free Sprint (15 m) 1 x 2	Free Sprint (10 m) 1x 2	Free Sprint (5 m) 2 x 4	Free Sprint (10 m) 1 x 4
		Alternate leg bounding (20m) 2x2	Free Sprint (15 m) 1 x 2	Alternate leg bounding(20m) 2x3		Alternate leg bounding (20m) 2x3	

### Soccer training program

The soccer training program was performed by players assigned to all three groups during six weeks with four sessions of soccer training per week. Each session lasted about 90 minutes including a warm-up, tactical work carried out with different types of possessions and small side games and ending with stretches of main muscle groups such as quadriceps, hamstring, hip flexors and calves. Two times per week, 15 to 20 minutes of aerobic capacity sessions were included. The training was supervised by the same experimenter (TP) and no additional strength or sprinting workout was allowed outside of the soccer practice established in each of the programs.

### Statistical analysis

All data are presented as mean ± standard deviation. In order to clearly assess the practical meaning of the results, data were analysed using the magnitude-based inference approach [32]. Changes in athlete scores were evaluated using effect sizes (ES) and 90% confidence limits. Within-group difference in pre and post-training of mechanical sprint properties and fascicle variables were assessed using standardised effect size (ES). The magnitude of the within-group changes was interpreted by using values of trivial (< 0.20), small (0.20 –< 0.60), moderate (0.60 –< 1.20), large (1.20 –< 2.00) and extremely large of the between-athlete variation at pre (i.e. smallest worthwhile change SWC). The probability that these differences actually exist was then assessed via magnitude-based qualitative inferences [33]. Qualitative inferences were based on quantitative chances of benefit outlined in [34]. Clinical chances are percentage chances that an observed effect is clinically positive/trivial/negative e.g. (40/40/20%) means an effect has 40% of chances to be positive, 40% to be trivial and 20% to be negative. Two separate statistical methods were used to assess the effectiveness of each method of training. To estimate inter-day reliability, intraclass correlation coefficient (ICC) and their 95% confidence intervals were calculated for variables related to biceps femoris architecture. Standar Error of Measurement (SEM) was calculated as the root mean square of total mean-square intrasubject variation. Pre- post-analysis was performed on each group’s data, to provide a clear effect of whether there were substantial and clear changes as a result of the training intervention. A second parallel group trials assessment compared the interventions. Probabilities that differences were higher than, lower than, or similar to the smallest worthwhile difference were evaluated qualitatively as possibly, 25% to 74.9%; likely, 75% to 94.9%, very likely, 95% to 99.5%; and most (extremely) likely, >99.5%.

Since the findings of present study could be used for athletes considered in isolation, individual analyses were performed to quantify for each variable and each group the number of responders and non-responders. Monitoring progression of an athlete with performance requires taking into account the magnitude of the SWC in performance and the uncertainty or noise in the test result [34], SWC being computed as one-fifth of the between-athlete standard deviation (a standardized or Cohen effect size of 0.20 [35]. Individual training responses were then considered as decrease (individual change < −1 SWC), trivial (from −1 SWC to +1 SWC) or increase (+1 SWC) for each variable if interest.

## Results

Mean ± SD values for all sprint performance and mechanical variables pre- and post-training intervention are shown for all groups in Table 2, along with within-group changes qualitative inferences (Table 3). Substantial differences were found in mechanical sprint variables in the “sprint group” with small to large changes with a *very likely* inference for all the mechanical outputs except for v_0_ (*possibly* inference). Contrastingly, these changes were less clear in “Nordic” and “soccer” groups, with small to trivial changes and *possibly* and *likely* inference post-training intervention.

**Table 2:** Sprint performance and mechanical output variables pre and post training for the control and intervention groups.

	NORDIC GROUP (n = 7)					
	Pre	Post	Post—*Pre*		*Inference*	
	$\bar{x}$ ± SD	$\bar{x}$ ± SD	%Δ ± SD	*ES; ±90% CL*		*Individual Response Increase/Trivial/Decrease*
*v*_0_ (m·s^-1^)	9.05 ± 0.24	9.04 ± 0.38	-0.11 ± 1.89	-0.03 ± 0.47	Trivial* *(neutral)*	***1–2–4***
*F*_0_ (N·kg^-1^)	6.85 ± 0.44	6.66 ± 0.28	-2.76 ± 3.99	-0.40 ± 0.45	Small** *(negative)*	***1–2–4***
*P*_max_ (W·kg^-1^)	15.4 ± 1.0	14.9 ± 0.6	-2.77 ± 4.43	-0.39 ± 0.46	Small** *(negative)*	***1–3–3***
*RF*_max_ (%)	46.9 ± 4.0	46.8 ± 2.3	-0.27 ± 5.13	-0.06 ± 0.60	Trivial* *(neutral)*	***3–1–3***
5 m (s)	1.42 ± 0.04	1.43 ± 0.03	1.02 ± 1.76	0.32 ± 0.41	Small* *(positive)*	***1–1–5***
20 m (s)	3.47 ± 0.07	3.50 ± 0.05	0.87 ± 1.59	0.35 ± 0.48	Small* *(positive)*	***2–1–4***
	SPRINT GROUP (n = 8)					
*v*_0_ (m·s^-1^)	8.91 ± 0.53	9.04 ± 0.55	1.46 ± 1.49	0.22 ± 0.16	Small* *(positive)*	***5–3–0***
*F*_0_ (N·kg^-1^)	6.49 ± 0.57	6.97 ± 0.63	7.42 ± 3.20	0.75 ± 0.22	Moderate*** *(positive)*	***8–0–0***
*P*_max_ (W·kg^-1^)	14.4 ± 1.8	15.7 ± 1.8	8.93 ± 3.16	0.64 ± 0.15	Moderate*** *(positive)*	***8–0–0***
*RF*_max_ (%)	44.3 ± 2.4	47.7 ± 2.5	7.80 ± 4.56	1.27 ± 0.49	Large*** *(positive)*	***8–0–0***
5 m (s)	1.46 ± 0.06	1.41 ± 0.06	-3.40 ± 1.43	-0.77 ± 0.22	Moderate*** *(negative)*	***8–0–0***
20 m (s)	3.55 ± 0.14	3.46 ± 0.14	-2.59 ± 1.05	-0.58 ± 0.16	Small*** *(negative)*	***8–0–0***
	SOCCER GROUP (n = 8)					
*v*_0_ (m·s^-1^)	8.95 ± 0.36	8.87 ± 0.38	-0.85 ± 2.12	-0.19 ± 0.30	Trivial* *(neutral)*	***3–1–4***
*F*_0_ (N·kg^-1^)	6.90 ± 0.79	7.01 ± 0.57	2.02 ± 5.38	0.12 ± 0.22	Trivial* *(neutral)*	***3–4–1***
*P*_max_ (W·kg^-1^)	15.3 ± 1.8	15.4 ± 1.4	1.08 ± 4.65	0.05 ± 0.21	Trivial** *(neutral)*	***1–6–1***
*RF*_max_ (%)	46.2 ± 4.6	48.0 ± 2.5	4.43 ± 7.07	0.35 ± 0.37	Small** *(positive)*	***6–0–2***
5 m (s)	1.42 ± 0.07	1.41 ± 0.05	-0.83 ± 2.20	-0.16 ± 0.27	Trivial*(negative)	***5–0–3***
20 m (s)	3.48 ± 0.14	3.48 ± 0.10	-0.18 ± 1.41	-0.05 ± 0.22	Trivial** *(positive)*	***3–1–4***

Values are mean ± standard deviation, percent change ± standard deviation and standardised effect size; ±90% confidence limits. Abbreviations: *n*, sample size; $\bar{x}$, mean; SD, standard deviation, %Δ, percent change; ES, effect size; 90% CL, 90% confidence limits; kg, kilogramme; *v*_0_, theoretical maximal velocity; m, metre; s, second; *F*_0_, theoretical maximal horizontal force; N, newton; *P*_max_, maximal power output; W, watt; *RF*_max_, maximal ratio of force after 0.3 seconds. Qualitative inferences are trivial (< 0.20), small (0.20 –< 0.60), moderate (0.60 –< 1.20) and large (> 1.20)

* possibly, 25 –< 75

** likely, 75 –< 95%

*** very likely, 95 –< 99.5. Positive, neutral and negative descriptors qualitatively describe the change between post and pre values and its importance relative to the specific variable.

**Table 3:** Sprint performance and mechanical variables comparisons of between-group post–pre changes.

	Post ‒ pre group change		Post ‒ pre group change		Post ‒ pre group change	
	Sprint Group–Soccer group		Sprint Group–Nordic group		Nordic Group–Soccer group	
	*ES; ±90% CL*	*Inference*	*ES; ±90% CL*	*Inference*	*ES; ±90% CL*	*Inference*
*v*_0_ (m·s^-1^)	*0*.*76 ± 0*.*96*	Moderate** *(positive)*	*0*.*26 ± 1*.*11*	Small* *(positive)*	*-0*.*38 ± 0*.*77*	Small* *(negative)*
*F*_0_ (N·kg^-1^)	*0*.*07 ± 0*.*78*	Trivial* *(positive)*	*1*.*18 ± 1*.*38*	Moderate** *(positive)*	*1*.*33 ± 1*.*19*	Large** *(positive)*
*P*_max_ (W·kg^-1^)	*0*.*41 ± 0*.*98*	Moderate** *(positive)*	*1*.*53 ± 1*.*40*	Large** *(positive)*	*0*.*97 ± 1*.*50*	Moderate** *(positive)*
*RF*_max_ (%)	*0*.*01 ± 1*.*01*	Trivial* *(positive)*	*0*.*38 ± 0*.*90*	Small* *(positive)*	*0*.*50 ± 0*.*85*	Small* *(positive)*
5 m (s)	*0*.*11 ± 0*.*21*	Trivial** *(neutral)*	*-1*.*10 ± 1*.*06*	Moderate** *(negative)*	*-0*.*99 ± 1*.*11*	Moderate** *(negative)*
20 m (s)	*-0*.*41 ± 1*.*01*	Small* *(negative)*	*-1*.*08 ± 1*.*26*	Moderate** *(negative)*	*-0*.*55 ± 1*.*40*	Small* *(negative)*

Values are mean ± standard deviation, percent change ± standard deviation and standardised effect size; ±90% confidence limits. Abbreviations: *n*, sample size; $\bar{x}$, mean; SD, standard deviation, %Δ, percent change; ES, effect size; 90% CL, 90% confidence limits; kg, kilogramme; *v*_0_, theoretical maximal velocity; m, metre; s, second; *F*_0_, theoretical maximal force; N, newton; *P*_max_, maximal power output; W, watt; *RF*_max_, maximal ratio of force after 0.3 seconds. Qualitative inferences are trivial (< 0.20), small (0.20 –< 0.60), moderate (0.60 –< 1.20) and large (> 1.20)

* possibly, 25 –< 75

** likely, 75 –< 95%. Positive, neutral and negative descriptors qualitatively describe the change between post and pre values and its importance relative to the specific variable.

**Fig 2:**
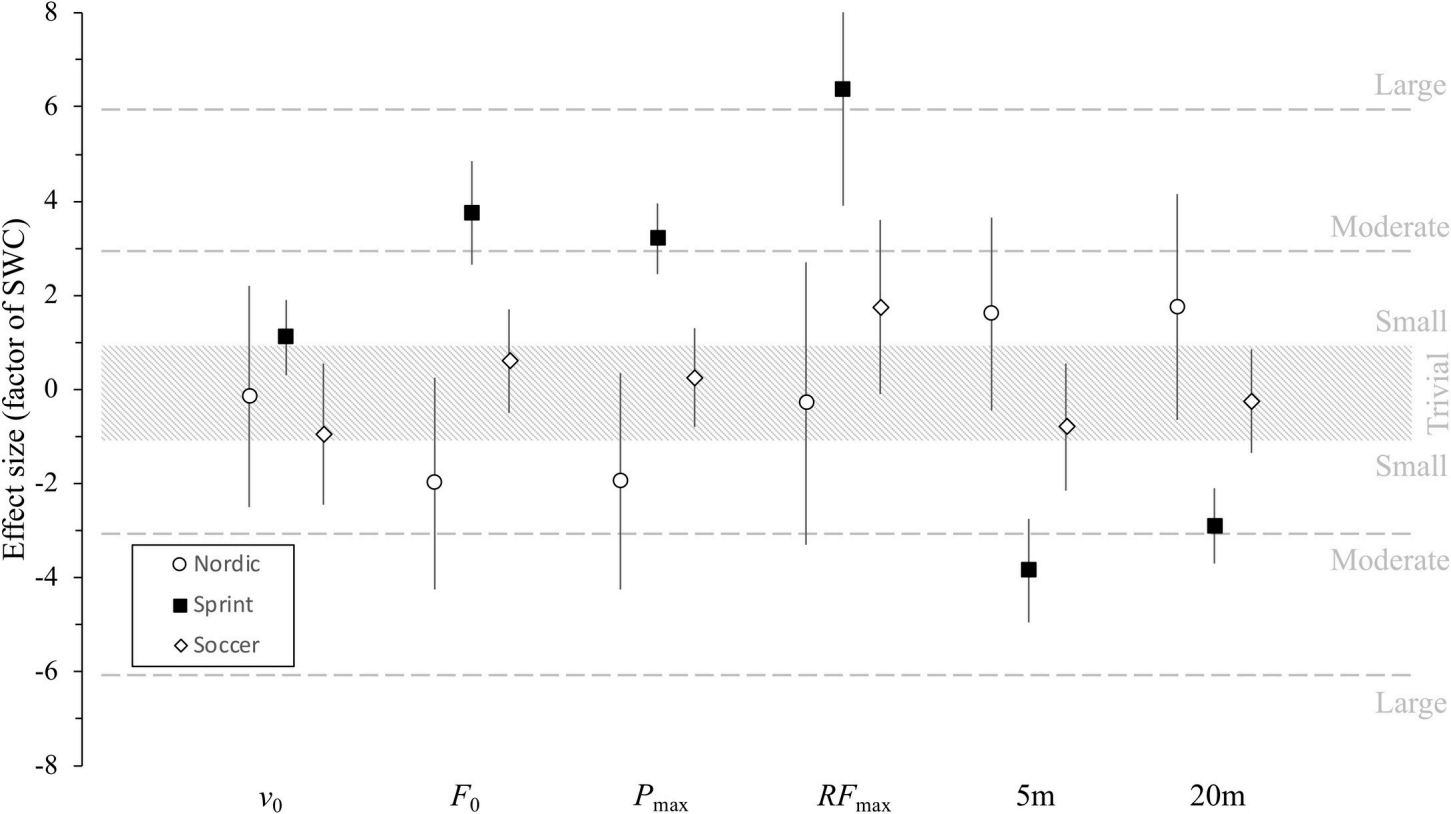
Magnitude of pre-post changes in the main sprint acceleration performance and mechanical outputs. The standardised differences are expressed as a factor of the smallest worthwhile change (SWC). Bars indicate the 90% confidence limits. v0: theoretical maximal velocity; F0: theoretical maximal horizontal force; Pmax: maximal power output; RFmax: maximal ratio of force; 5 m: 5 m sprint time; 20 m: 20 m sprint time.

The “sprint group” showed a small increase in v_0_ (*possibly*) and RF_max_ (*very likely*), moderate in F_0_ (*very likely*), 5 m and 20 m times (*very likely*, respectively), and large in Pmax (*very likely*), whereas the “nordic group” showed changes ranging between small and very large increase interaction (Table 3). Trivial and unclear changes were observed in most of the sprint mechanical variables analyzed between “sprint group” and “soccer group” except for v_0_ and Pmax with small and moderate changes observed. Similar changes were observed between “Nordic” and “soccer” groups with small changes in v_0_, RF_max_ and 20 m time with *possibly* to *likely* inference, and moderate changes in P_max_ and 5 m time with *likely* inference, meanwhile a large change was observed in F_0_ with *likely* inference (Table 3, Fig 2).

Furthermore, variables related to biceps femoris architecture such as fascicle length, pennation angle and muscle thickness pre- and post-training intervention are shown for all groups in Table 4, along with within-group changes qualitative inferences (Table 5). The ICC for variables related to biceps femoris architecture were 0.989 (0.959–0.998) for fascicle length, 0.964 (0.865–0.993) for pennation angle and 0.981 (0.929–0.996) for muscle thickness. SEM ranged from 1.68% to 2.83%. ICC was calculated with 7 sport sciences students in similar conditions (as described in Material and Methods section; 5 min rest and lying down before the measurement) separated by 24 hours.

**Table 4:** BFlh muscle architectural variables pre and post training for the control and intervention groups.

	NORDIC GROUP (n = 7)					
	Pre	Post	Post—Pre		*Inference*	
	$\bar{x}$ ± SD	$\bar{x}$ ± SD	%Δ ± SD	*ES; ±90% CL*		*Individual Response Beneficial/Trivial/Harmful*
FL-2legs Mean (cm)	9.93 ± 1.10	10.66 ± 1.01	7.38 ± 4.03	0.58 ± 0.33	Small** *(positive)*	***5–1–1***
PA-2legs Mean (°)	13.29 ± 2.61	14.52 ± 1.84	9.24 ± 8.60	0.41 ± 0.62	Small*(positive)	***4–2–1***
Thickness BFlh 2legs (cm)	2.28 ± 0.22	2.40 ± 0.19	5.04 ± 2.11	0.46 ± 0.40	Small**(positive)	***4–1–2***
	SPRINT GROUP (n = 8)					
FL-2legs Mean (cm)	10.23 ± 1.91	11.89 ± 1.16	16.21 ± 10.26	0.77 ± 0.67	Moderate** *(positive)*	***7–0–1***
PA-2legs Mean (°)	14.19 ± 1.82	14.26 ± 1.57	0.49 ± 2.47	0.03 ± 0.34	Trivial* *(neutral)*	***4–2–2***
Thickness BFlh 2legs (cm)	2.39 ± 0.16	2.52 ± 0.09	5.80 ± 2.11	0.76 ± 0.47	Moderate*** *(positive*	***5–3–0***
	SOCCER GROUP (n = 8)					
FL-2legs Mean (cm)	10.20 ± 1.08	10.17 ± 0.82	0.31 ± 1.69	-0.03 ± 0.26	Trivial** *(negative)*	***1–2–5***
PA-2legs Mean (°)	12.47 ± 1.60	12.61 ± 1.60	1.12 ± 3.00	0.08 ± 0.34	Trivial* *(positive)*	***2–4–2***
Thickness BFlh 2legs (cm)	2.22 ± 0.23	2.25 ± 0.21	1.43 ± 1.98	0.12 ± 0.31	Trivial*(positive)	***2–4–2***

Values are mean ± standard deviation, percent change ± standard deviation and standardised effect size; ±90% confidence limits. Abbreviations: *n*, sample size; $\bar{x}$, mean; SD, standard deviation, %Δ, percent change; ES, effect size; 90% CL, 90% confidence limits; FL-2legs Mean, fascial length mean for right and left legs; PA-2legs Mean, pennation angle mean for right and left legs; Thickness BFlh 2 legs, Thickness BFlh mean for right and left leg. Qualitative inferences are trivial (< 0.20), small (0.20 –< 0.60) and moderate (0.60 –< 1.20)

* possibly, 25 –< 75

** likely, 75 –< 95%

*** very likely, 95–<99.5. Positive, neutral and negative descriptors qualitatively describe the change between post and pre-values and its importance relative to the specific variable.

**Table 5:** BFlh muscle architectural variables between-groups comparisons of post–pre changes.

	Post ‒ pre group change		Post ‒ pre group change		Post ‒ pre group change	
	Sprint Group–Soccer group		Sprint Group–Nordic group		Nordic Group–Soccer group	
	*ES; ±90% CL*	*Inference*	*ES; ±90% CL*	*Inference*	*ES; ±90% CL*	*Inference*
FL-2legs Mean (cm)	*2*.*13 ± 0*.*96*	Large**** *(positive)*	*1*.*16 ± 1*.*18*	Moderate** *(positive)*	*-0*.*26 ± 0*.*91*	Small* *(negative)*
PA-2legs Mean (°)	*1*.*11 ± 0*.*94*	Moderate** *(positive)*	*-0*.*11 ± 0*.*99*	Trivial* *(negative)*	*-0*.*86 ± 0*.*97*	Moderate** *(negative)*
Thickness BFlh 2legs (cm)	*1*.*14 ± 0*.*74*	Moderate*** *(positive)*	*0*.*55 ± 0*.*46*	Small** *(positive)*	*-0*.*57 ± 0*.*98*	Smal*l* *(negative)*

Values are mean ± standard deviation, percent change ± standard deviation and standardised effect size; ±90% confidence limits. Abbreviations: *n*, sample size; $\bar{x}$, mean; SD, standard deviation, %Δ, percent change; ES, effect size; 90% CL, 90% confidence limits; FL-2legs Mean, fascial length mean for right and left legs; PA-2legs Mean, pennation angle mean for right and left legs; Thickness BFlh 2 legs, Thickness Biceps Long Femoris Head mean for right and left leg. Qualitative inferences are trivial (< 0.20), small (0.20 –< 0.60), moderate (0.60 –< 1.20) and large (>1.20)

* possibly, 25 –< 75

** likely, 75 –< 95%

*** very likely, 95–<99.5

**** most likely, >99.5. Positive, neutral and negative descriptors qualitatively describe the change between post and pre values and its importance relative to the specific variable.

Substantial changes were observed in fascicle length, pennation angle and muscle thickness for “nordic group” with a *possibly* and *likely* inference after training intervention. Similarly, to “nordic group”, the “sprint group” showed substantial changes in fascicle length and muscle thickness with moderate changes with a *likely* and *very likely* inference after training intervention, whereas a trivial effect was observed for pennation angle in this group. Finally, in the “soccer group”, trivial changes were reported with a *possibly and likely* inference after the training intervention for all variables.

**Fig 3:**
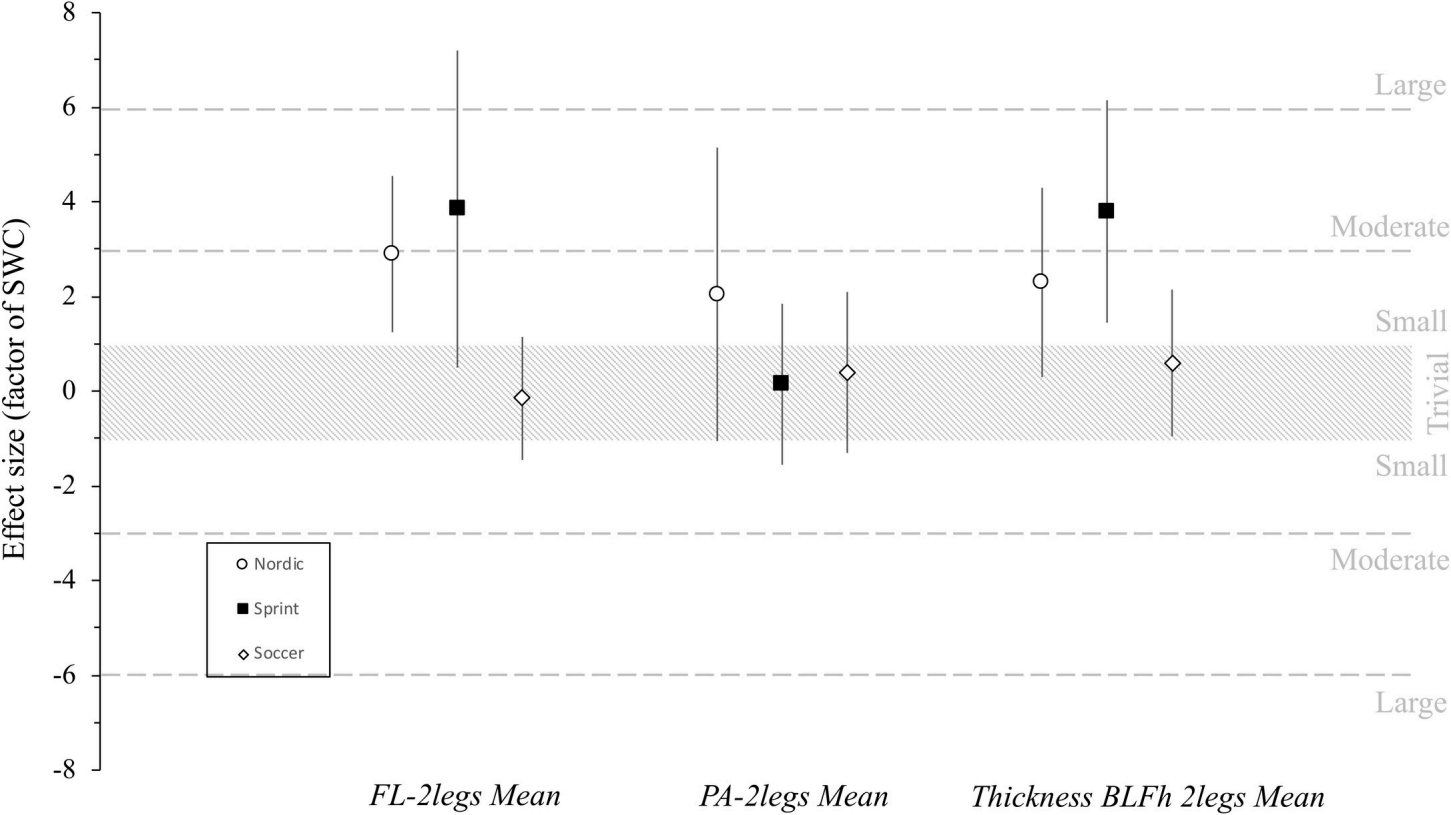
Magnitude of pre-post changes in the main BFlh muscle architectural variables. The standardised differences are expressed as a factor of the smallest worthwhile change (SWC). Bars indicate the 90% confidence limits. FL-2legs: fascial length means for right and left legs; PA-2legs: pennation angle mean for right and left legs; Thickness BLFh 2 legs: Thickness Biceps Long Femoris Head mean for right and left leg.

The between-group comparison showed that the “sprint group” had a *likely* moderate (fascicle length), a *possibly* trivial (pennation angle) and *likely* small (muscle thickness) changes in biceps femoris architectural variables in comparison with “nordic group” (Table 5, Fig 3). Similarly, *most likely* large (fascicle length), a *likely* moderate (pennation angle) and *very likely* moderate (muscle thickness) changes were found between “sprint” and “soccer” groups. “Nordic group” showed a *possibly* small change (fascicle length), a *likely* moderate (pennation angle) and a *likely* small change (muscle thickness) compared to soccer group (Table 5).

## Discussion

The aim of this study was to compare the effects of two 6-week training programs added to the normal soccer training: eccentric hamstring strength using the nordic hamstring exercise versus a comprehensive sprint training program, on biceps femoris long head architecture, sprint acceleration performance mechanical outputs. The main findings were (a) the addition of two weekly sessions of sprint training to regular soccer practice induced moderate improvements in biceps femoris long head fascicle length compared to the small increases showed after isolated eccentric strength training or not changes when practicing soccer training alone; (b) biceps femoris muscle pennation angle showed a small increase only when nordic hamstring exercise was added to soccer training, and not in the case of sprint training; (c) sprinting training added to regular soccer training produced small to large improvements in both sprint acceleration performance and the underlying mechanical outputs (except maximal running velocity), which contrasts with trivial or even small negative changes in the case of hamstring eccentric strength training or when practicing soccer training.

This study is the first to explore the architectural and morphological adaptations of the hamstrings in response to isolated knee flexor eccentric strength training versus sprint training programmed in addition to regular soccer training during pre-season, and not in isolated conditions without concomitant sport practice [8,11,36,37]. We think this point is an important feature of the current study, since it is more in line with the real sport practice, where soccer players train for soccer first, and any type of intervention is added to the basic sport practice. By definition, any complementary intervention comes in addition to the main sport practice, so it should not be studied separately. It is important to keep this more realistic scenario in mind when discussing the results of this study and previous works, since it may influence the practical conclusions of these works.

### Sprint and eccentric exercises induce changes in BFlh fascicle length

Collectively, although our results suggest that both types of training added to soccer practice induced increases in the length of the fascicle BFlh, the sprint showed moderately superior adaptations (16%) compared to the NHE (7%). These adaptations may result from the addition of in-series sarcomeres [10]. It has been proposed that this increase in serial sarcomeres is associated with both a rightward shift in a muscle’s force-length relationship, while also reducing its susceptibility to damage associated with strain [10]. However, fascicle lengthening due to increases in tendon stiffness is one possible alternative explanation [38]. Due to the limitations of the static method used in the present and previous studies[12,13], further research is clearly needed to fully understand the mechanism(s) responsible for these architectural changes (muscle-tendon interaction) and validate the suggested hypotheses in dynamic (not only isolated, static) actions.

The greater increase of BFlh FL observed after a comprehensive sprinting programme compared to NHE could be related to the continuous and increased intensity lengthening of the muscle-tendon unit induced by high-speed movements (for exercises targeting the velocity end of the sprint force-velocity spectrum) and sprint-specific strength overload (for exercises targeting the force-end of the spectrum). Similarly, studies on the architecture of other muscle groups, such as the vastus lateralis reported a significant greater increase in fascicle length without pennation angle changes (as in the present study) after a period of sprint/jump training alone compared to those who added different resistance training programs. [39]. Recently, sprint training has been shown effective for improving eccentric hamstring strength in adolescent athletes, in addition to the positive effects on sprint performance [40]. These arguments, among others, may partly explain recent preliminary research suggesting a protective effect of an adequate exposure to maximum velocity sprint efforts in different soccer codes that would place sprinting itself as part of a comprehensive strategy (including other evidenced strategies) to prevent soft-tissue injuries [41,42].

The observed increase in BFlh fiber length at rest after NHE was added to regular soccer training in the present study (7%) is similar to the results found in a group of elite young French soccer players following an eccentric-biased hamstring training program (~ 5%) [43] but considerably lower than the increases (between 16–32%) observed in other studies after 6–12 weeks of various strength training modalities (leg curl, isokinetic dynamometry and NHE, respectively) focused on knee flexor eccentric overload [11,36,37]. Even that architectural variables vary considerably between individuals, which may explain that the average changes in architectural variables differ between studies, the ultrasonography approach used [11,36,37,44] to extrapolate the changes in FL may also justify the large difference in FL changes observed between the current study and previous ones [11,36,37,44]. The static-image sonographic technique used to estimate the heterogeneous and non-uniform biceps femoris fascicle architecture presents clear limitations [12,13] that could affect the results obtained in studies, including the current one. Nonetheless, the method used in this study, manual linear extrapolation, has recently been recommended due to a lower fascicle estimation and greater accuracy with respect to trigonometric equation methods used in other studies if only conventional ultrasound imaging is available [30]. Thus, on this specific point, the present results, as those of previous studies, should be taken with caution until more research on the improvement of ultrasonographic approach for fascicle length measurement is available.

The discrepancy in results could also be related to the possibility that, in contrast with the more realistic training integrated to the usual soccer practice proposed in the present study [43,45], other studies included isolated eccentric training contractions targeting an increase in fascicle length, but not concomitant with the usual soccer practice, and the associated specific movements patterns [11,36,37]. Based on the fact that soccer training alone induced increases in posterior thigh muscles concentric strength (but not eccentric) [14] and that different studies reported a decrease in fascicle length after concentric training [8,9], the present results suggest that soccer practice may mitigate the increase in FL associated with isolated eccentric contractions when these two interventions are programmed together, which is almost always the case in real training contexts.

Fascicle lengthening is one possible mechanism by which the NHE and other eccentric or long-length hamstring exercises have been proven effective on hamstring injury reduction. Timmins et al. [11] recently showed, prospectively, that professional soccer players with average BFlh fascicle length <10.56 cm were ∼4 times more likely to suffer a hamstring strain than athletes with longer fascicle length and that the probability of injury was overall estimated to decrease by ∼74% on average for every 0.5 cm increase in fascicle length. In the current study, we observed increases in BFlh fascicle length of ∼0.7 cm after Nordic and ∼1.6 cm after Sprint training programs added to the regular soccer practice. This would, according to the proportions described earlier, likely result in a higher reduction in hamstring injury risk after Sprint than Nordic interventions. However, although theoretically longer fascicles may enhance sprint performance by altering the operating range of muscles and increasing muscle force-generating capacity, only the Sprint intervention induced clear improvements in sprint performance and mechanics, which was not the case of the Nordic training program, as discussed below.

Regarding BFlh pennation angle, this study confirms previous findings by Lovell et al. [45] showing that the addition of knee flexor eccentric training through NHE post soccer training resulted in a similar hypertrophic response, identified by an increased pennation angle and muscle thickness (PA ~10%). This increase is considered to represent an increase of the physiological cross-sectional area with more myofibrils in parallel, enabling the improved transmission of force developed through the muscle-tendon unit, and in turn a higher architectural gear ratio [7,46]. The latter will allow the pennate biceps femoris to limit the strain experienced by active fascicles and provide some degree of protection during fast-velocity lengthening actions [46]. Mechanical tension and intramuscular metabolic stress, determine the hypertrophic signal of the muscle that may be amplified (as in this study) when resistance training was performed following high-intensity interval and endurance training, which has also been shown to trigger anabolic signaling pathways and hypertrophy [7].

### Greater improvements of sprint performance and mechanics after sprint training

Concerning the sprint mechanics and performance outcomes, the ability to produce high acceleration and speed is considered an important quality for performance in soccer [1]. Although hamstrings play a role in the forward orientation of the ground reaction force, especially at high running speed (when their torque capability and electrical activity are both considered), the results of this study showed no benefits (and even small negative changes) of a NHE force program added to regular soccer practice on sprint mechanical outputs and performance. This is consistent with previous results [14], and contrasts with two very similar recent studies reporting small to moderate improvements (with high inter-individual variability) in sprint performance after NHE training in a group of soccer players [15,16]. Although sprint acceleration mechanical properties were not analyzed in these studies [15,16], the time of realization of the program (pre-season versus in-season), and match and training high running speed exposure, internal and external training load may explain the differences between studies. Ishøi et al. [15] reported concomitant average group changes, but not the direct association, on an individual basis, between improvements in knee flexors strength and sprint performance. Furthermore, previous studies [14] reported substantial increases in hamstring strength with no or minimal concomitant changes in sprint acceleration mechanical outputs and performance. These two facts clearly question the idea that isolated posterior thigh strengthening alone (using for example the NHE modality) directly results in the improvement of such a complex neuromuscular task as maximal sprint acceleration, despite the fact that hamstring muscles play a role, especially when taking their sprint-specific activity as derived from electromyographical analysis [17]. This is not surprising given the large differences in the timing, velocity, length, hip-knee kinematics and overall intra- and inter-muscles coordination between any isolated hamstring strengthening exercise (a fortiori a single-joint, bilateral one like the NHE) and the sprint acceleration tasks.

In contrast, the specific comprehensive sprint program used in the present study resulted in moderate and very likely improvements on both sprint mechanics (mainly acceleration variables) and performance after 6 weeks during soccer preseason where the total workload is greater than in-season [47], comparatively favoring the increase of aerobic fitness to the detriment of anaerobic capacity [48]. Specifically, this program (likely due to the use of resisted and unresisted sprints) resulted in higher maximal force output in the horizontal direction (*F*_0_ i.e. first meters of the acceleration phase), also evidenced by a greater RFmax. This specific variable of the sprint force-velocity profile, which was shown to markedly decrease after hamstring injury [18], increased here substantially in the Sprint group, whereas only a small increase was observed in the ability to produce horizontal force at high speeds (v_0_). The results of the Sprint group are similar to what Morin et al. [49] reported in a pilot study using very heavy sled resistance training in soccer players: specific *F*_0_ and RF Max improvements with only trivial effect on v_0_, and only trivial changes in the control group who performed unresisted sprints. Interestingly, this brings support to the use of a comprehensive sprint training that (a) covers/stimulate a large spectrum of force and velocity conditions (ranging from heavy resistance to flying start sprints) and (b) uses acceleration athletics drills and horizontal plyometric exercises. We think that this represents a more comprehensive (thus potentially more effective) overload than using unresisted sprints alone. Furthermore, soccer practice itself already includes unresisted, “classical” sprints, by definition. Our results suggest that this type of multifaceted sprint-specific program is effective to counteract the decrease in *F*_0_ observed after soccer preseason [50] or hamstring injury [18]. Although it is still a hypothesis, a reverse thinking would make this increase in maximal sprint force output potentially beneficial in the return-to-performance, or even prevention process. This should be the topic of future studies, but it makes sense in light of the data previously published on the topic and the current results.

Some limitations associated with this manuscript should be acknowledged. No collection of match and training exposure, internal and external training load variables was performed during the study. Although all these variables were likely very similar among players, they are possibly confounding factors of fascicle length adaptation. With that being said, the present study is, to our knowledge, the first to integrate and compare, within the same randomized controlled protocol, a commonly used injury prevention method (using an isolated, single-joint hamstring strength exercise, NHE), and a comprehensive training program specifically targeting sprinting performance (through stimulation of the entire force-velocity spectrum) in a realistic soccer practice context.

Secondly, the use of two-dimensional ultrasound to estimate fascicle length, although previously validated against cadaveric measurements [51], has some associated methodological limitations mainly derived from a reduced field of view as a consequence of a too small transducer width resulting in a greater fascicle estimations, restricted region of interest analyzed, questionable mathematical extrapolations and omission of fascicle and aponeurosis 3-dimensional curvature [12,30,52,53]. Moreover, the fact that the assessment method is based on 2-dimensional static measurement conditions limits its extrapolation to dynamic tasks. Future research should consider the use of extended field of view ultrasonography and 3-dimensional measurements to minimize potential error and take into account fascicle rotation during dynamic conditions, beyond resting, static conditions [51–53]. Finally, we could not assess the hamstring force output in the current study (via for example isokinetic or Nordic exercise testing), but a recent study showed that a sprint program induced some positive changes in both sprint performance and hamstring eccentric strength output as measured during the Nordic exercise in young athletes [40].

## Conclusions

Assuming fascicle length as a factor to be considered in the management of hamstring strain injuries, adding a sprint-focused program to regular soccer training induced greater increases in biceps femoris fascicle length than incorporation of Nordic hamstring exercise as a complementary intervention during the first 6 week of preseason period, compared with soccer practice alone. However, only the sprint comprehensive training provided this potentially preventive stimulus (increase fascicle length), and at the same time induced better sprint performance and mechanical outcomes, which could be considered a practical “win-win” strategy for the management of hamstring injuries. Due to the specificity of sprint training and low cost of testing methods (all variables can now be derived from split times or slow-motion videos with a validated App [54] of the sprints and online free computation spreadsheets (https://www.researchgate.net/publication/321767606_Spreadsheet_for_Sprint_acceleration_force-velocity-power_profiling), both players and staff can be more compliant to this type of intervention, compared to previously proposed methods. Finally, based on the current pilot results, further studies should test whether a comprehensive sprint training offers significant injury prevention advantages, as suggested recently [41,42].

## Supporting information

S1 Raw data (XLSX)
